# Mitral Annular Plane Systolic Excursion (MAPSE): A Review of a Simple and Forgotten Parameter for Assessing Left Ventricle Function

**DOI:** 10.3390/jcm13175265

**Published:** 2024-09-05

**Authors:** Liviu Cirin, Simina Crișan, Constantin-Tudor Luca, Roxana Buzaș, Daniel Florin Lighezan, Cristina Văcărescu, Andreea Cozgarea, Cristina Tudoran, Dragoș Cozma

**Affiliations:** 1 Cardiology Department, “Victor Babes” University of Medicine and Pharmacy, 2 Eftimie Murgu Sq., 300041 Timisoara, Romania; cirin.liviu@umft.ro (L.C.); constantin.luca@umft.ro (C.-T.L.); cristina.vacarescu@umft.ro (C.V.); andreea.cozgarea@umft.ro (A.C.); dragos.cozma@umft.ro (D.C.); 2 Research Center of the Institute of Cardiovascular Diseases Timisoara, 13A Gheorghe Adam Street, 300310 Timisoara, Romania; 3 Institute of Cardiovascular Diseases Timisoara, 13A Gheorghe Adam Street, 300310 Timisoara, Romania; 4 Department of Internal Medicine I, “Victor Babes” University of Medicine and Pharmacy, Eftimie Murgu Square, No. 2, 300041 Timisoara, Romania; buzas.dana@umft.ro (R.B.); dlighezan@umft.ro (D.F.L.); 5 Center for Advanced Research in Cardiovascular Pathology and Hemostaseology, “Victor Babes” University of Medicine and Pharmacy, 300041 Timisoara, Romania; 6 County Clinical Emergency Hospital Sibiu, 550245 Sibiu, Romania; 7 Department VII, Internal Medicine II, Discipline of Cardiology, University of Medicine and Pharmacy “Victor Babes” Timisoara, E. Murgu Square, Nr. 2, 300041 Timisoara, Romania; tudoran.cristina@umft.ro; 8 County Emergency Hospital “Pius Brinzeu”, L. Rebreanu, Nr. 156, 300723 Timisoara, Romania; 9 Center of Molecular Research in Nephrology and Vascular Disease, Faculty of the University of Medicine and Pharmacy “Victor Babes” Timisoara, E. Murgu Square, Nr. 2, 300041 Timisoara, Romania

**Keywords:** MAPSE, LVEF, echocardiography, review

## Abstract

Mitral annular plane systolic excursion (MAPSE) was a widely used and simple M-mode echocardiographic parameter for determining the left ventricle (LV) longitudinal systolic function. The purpose of this review is to analyze the use of MAPSE as a simple LV systolic function marker in different clinical scenarios, especially given the recent paradox of choices in ultrasound markers assessing cardiac performance. Recent data on the use of MAPSE in the assessment of LV function in different settings seem to be relatively scarce, given the wide variety of possible causes of cardiovascular pathology. There remain significant possible clinical applications of MAPSE utilization. This review included all major articles on the topic of mitral annular plane systolic excursion published and indexed in the PubMed, Google Scholar, and Scopus databases. We analyzed the potential implications of using simpler ultrasonographical tools in heart failure diagnosis, prediction, and treatment. MAPSE is a dependable, robust, and easy-to-use parameter compared to ejection fraction (EF) or global longitudinal strain (GLS) for the quick assessment of LV systolic function in various clinical settings. However, there may be a gap of evidence in certain scenarios such as conventional cardiac pacing.

## 1. Introduction

Mitral annular plane systolic excursion (MAPSE) is an M-mode-derived ultrasound parameter for assessing left ventricle (LV) systolic longitudinal function. It is a simple and reproducible index that correlates well with left ventricular ejection fraction (LVEF) and is a clinically established echocardiographic parameter that can be used in various stages of cardiovascular disease [1]. Even though it has long been superseded by other, more modern and complex parameters and techniques, such as tissue Doppler imaging, strain-rate imaging, or three-dimensional echocardiography, its appeal still consists in its ease of use and ready availability. The LV is conical in shape on the longitudinal section, with a central cavity surrounded by muscular walls, while, in a transverse section, it more resembles a circle. The LV wall is composed of three muscular “layers”, which, based on their alignment, are a subepicardial layer, a middle layer, and a subendocardial layer. These three layers also seem to be differently oriented, thus giving birth to oblique muscle fibers in the subepicardium, circumferential in the middle layer, and longitudinal in the subendocardium [2,3]. This specific anatomical arrangement and the mechanical motion imply that, during the cardiac cycle, certain ultrasound parameters can be used for approximating LV function [4]. Several techniques and parameters have been used for the assessment of LV function by two-dimensional cardiac ultrasound. The most widely used, documented, and currently recommended by both the European Association of Cardiovascular Imaging (EACVI) and the American Society of Echocardiography (ASE) for the assessment of LVEF, is the modified Simpson’s rule (biplane method of disks). Even though LVEF is a robust and reliable indicator of left ventricle systolic function, it does have its pitfalls. Due to the geometrical assumptions made by using the Simpson method, the accurate measurement of the LVEF requires good endocardial border tracing in end-systole and end-diastole and an adequate-quality image in the apical windows. These tracings eventually divide the LV cavity into a predetermined number of disks (usually 20), and since tracing of the entire LV cavity border is not achievable, some geometric assumptions need to be made.

**Figure 1:**
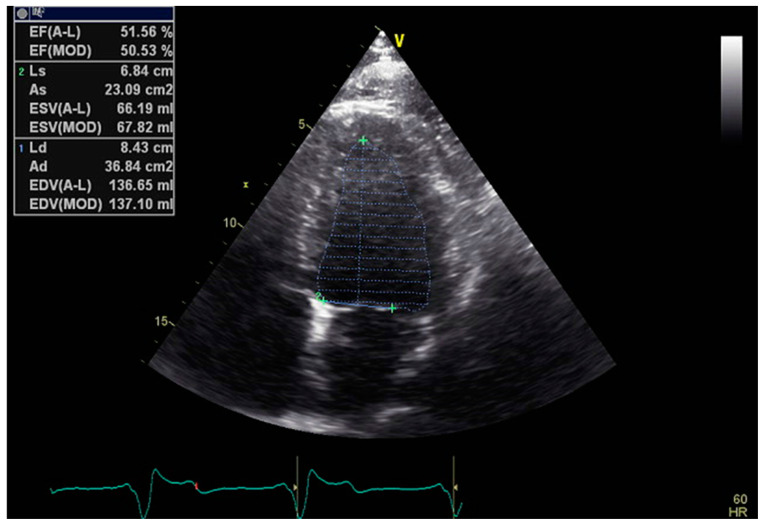
Example of 2D LVEF determination in the A4C view.

The monoplane Simpson method requires the tracing of endocardial borders just in the apical four chambers (A4C) view, while the more precise and the preferred one, the biplane method, already requires high-quality images in two distinct views, the A4C and the apical two chamber (A2C) [5] (Figure 1).

**Figure 2:**
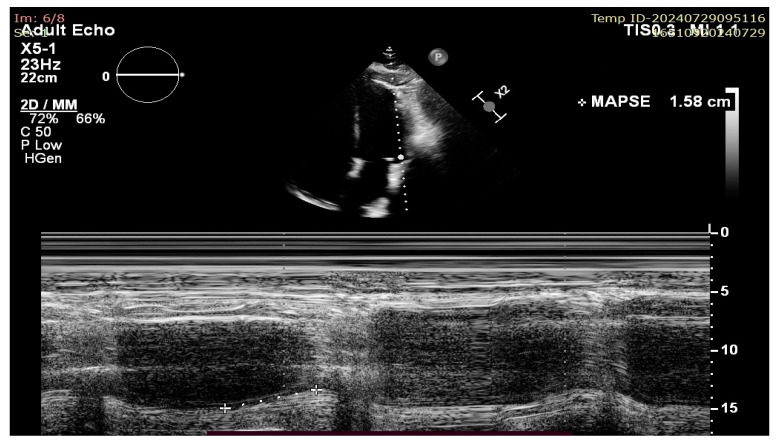
Lateral MAPSE measurement in the apical four chamber view.

More modern ultrasound techniques such as two-dimensional speckle tracking echocardiography (2D-STE) with global longitudinal strain (GLS) measurement and 3D ejection fraction (3D-EF) provide excellent accuracy and, at least in the case of 3D-EF, correlate well with CMR-derived volumes and results. However, all of these techniques share the following common disadvantages: they are time consuming, operator dependent, rely on image quality, and/or require more complex and expensive ultrasound machines. On the other hand, MAPSE measurement does not require good image quality (because it is a linear measurement that is less affected by artefacts) or multiple views and is not a time-consuming technique, thus making its measurement extremely useful in case of emergency settings and poor sonographic windows [6]. There has historically been ample data published on its usefulness in evaluating the longitudinal systolic function of the LV in heart failure (HF); however, recent publications have raised the question of its validity and use as a sensitive marker of early LV dysfunction and as a prognostic tool not only for HF patients, but also in the setting of septic shock, cardio-oncology, and many others [7,8]. There appears to be a lack of validation of its use in the context of conventional, endocardial right ventricular pacing (Figure 2).

The purpose of this article was to review the current, more recently available evidence on the use of MAPSE as a marker of cardiac function in different scenarios, as well as to offer an overview of its applications in day-to-day clinical situations.

## 2. Materials and Methods

**Figure 3:**
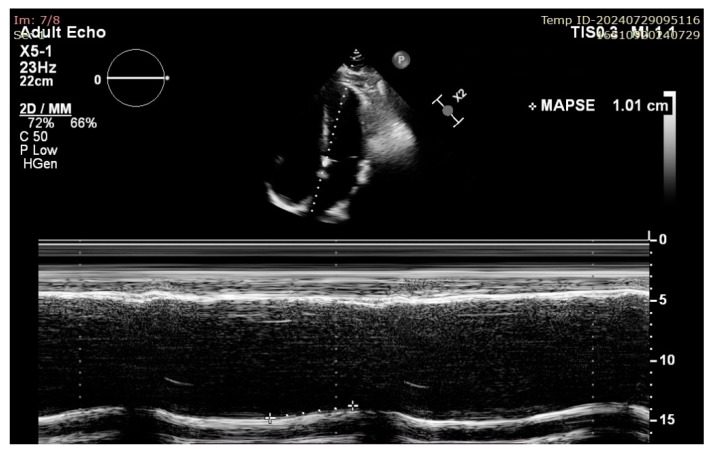
Example of septal MAPSE measurement in A4C view in a patient with impaired LV longitudinal function.

We performed a literature search for different types of published studies (retrospective, prospective, and reviews) using the PubMed, Google Scholar, and Scopus databases with the keywords “MAPSE” OR “MITRAL ANNULAR PLANE SYSTOLIC EXCURSION”. The selection of studies for this review followed the PRISMA (Preferred Reporting Items for Systematic Reviews and Meta-Analyses) guidelines. We performed manual searches and used MeSH (Medical Subject Headings) terms on PubMed to identify articles published on MAPSE. We excluded from selection articles not written in English, articles with subjects other than humans, articles with CMR-derived MAPSE, publications with only abstracts available, and duplicate entries. The search was performed in February 2024, with no publishing year restriction, by experienced cardiologists with expertise in echocardiography. We performed a manual initial search for articles using the specific keywords “MAPSE” and “MITRAL ANNULAR PLANE SYSTOLIC EXCURSION” in the title. In addition, using the MeSH term option available in PubMed, we conducted another search with the following terms: ((“MAPSE” [Mesh])). All eligible and chosen articles were organized into a Microsoft Excel table (version 2408), which included columns for the article’s title, authors, year of publication, journal, and publication type (Figure 3).

**Figure 4:**
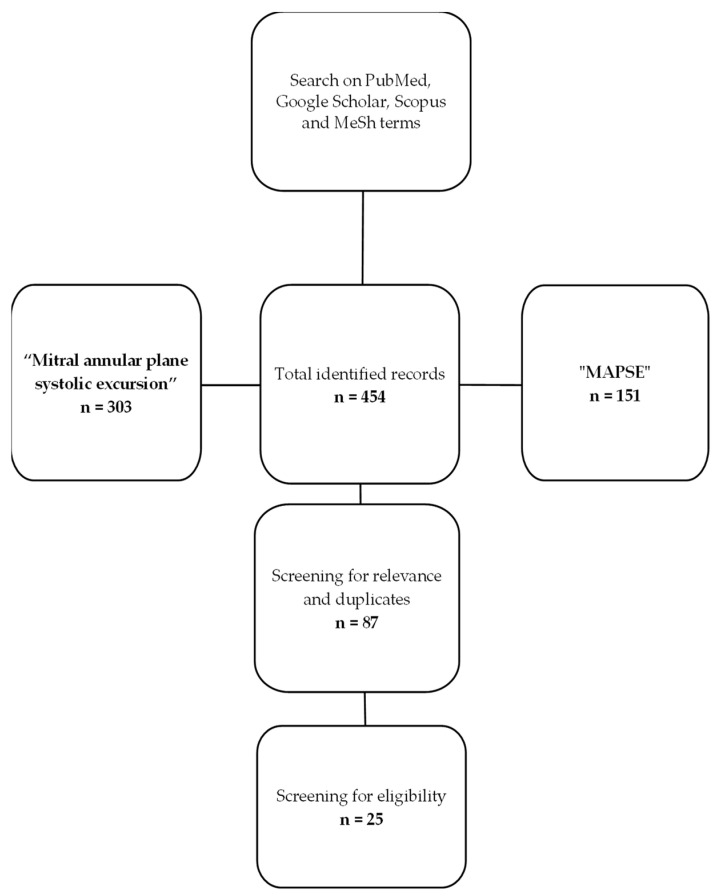
Flow diagram of the review process.

The diagram below exemplifies the methodology used by the authors for article selection and was created using Microsoft Office suite. We have found a total of 454 articles published and indexed in the search engines with the keywords of “mitral annular plane systolic excursion” and “MAPSE”. Out of the total number of identified records, we discarded duplicates and articles that were not relevant or did not correspond to the inclusion criteria set at the beginning, with the final number being 25 articles (Figure 4).

## 3. Results

We have included in the table below a selected number of relevant articles on the subject published in the last decade, having specified the title, authors, year of publication, number of patients included, and conclusions (Table 1).

### 3.1. Heart Failure

MAPSE has a strong correlation with LVEF, which has been validated in multiple studies over time, being a valuable tool for HF screening in the mid-range and reduced phenotypes [25]. It also seems that this correlation is maintained over the whole range of HF types, with one meta-analysis discovering that MAPSE is a more sensitive and specific indicator of LV systolic function in patients with heart failure with preserved ejection fraction (HFpEF) compared to LVEF and a good predictor of mortality and risk of long-term survival in patients diagnosed with HF [26]. As far as HFpEF is concerned, an article published in 2011 by Wenzelburger et al. indicates that MAPSE correlates well with other, more complex measurements of ventricular function in this subgroup of patients at rest and during exercise, being a useful tool for HFpEF diagnosis, especially during treadmill exercise testing using a modified Bruce protocol [27]. Another study performed by Elnoamany M.F. et al. showed a significative negative correlation between MAPSE and brain natriuretic peptide (BNP) levels [28]. There also appears to be an increase in right atrial (RA) dyssynchrony (assessed as the so-called PA′-TDI interval) in HFpEF patients with reduced MAPSE values, which suggests that these patients might be at an increased risk of developing arrhythmias [20]. Left atrioventricular plane displacement (AVPD), commonly measured ultrasonographically as the MAPSE index, is related to both systolic and diastolic LV function [29].

### 3.2. Hypertensive Heart Disease

In hypertensive patients, especially those with concentric LV hypertrophy, there is a deterioration of longitudinal function [30]. The determination of LV longitudinal function by the measurement of left atrioventricular plane displacement using M-mode ultrasound is reliable and reproducible. Longitudinal function impairment has been demonstrated to have prognostic value and is an independent cardiovascular risk marker in these patients, as proven by data in the literature [31,32].

### 3.3. Ischemic Heart Disease

Several publications have reported on the correlation between MAPSE and LV systolic function in patients with ischemic heart disease. An article published in 2018 by Magdy G et al. on 50 patients with ischemic heart disease and HFrEF reports that MAPSE can be an easy tool for assessing contractile reserve before revascularization, showing a significant positive correlation with LVEF [15]. The literature also cites left atrioventricular plane displacement as a clinically useful tool in patients with stable coronary artery disease and an independent prognostic tool in these patients not influenced by previous myocardial infarction [33].

### 3.4. Aortic Stenosis

We do know that, in left-sided valvular heart disease, MAPSE is affected by localized wall motion abnormalities around the area of the mitral valve, by severe mitral annular calcification, and by mitral valve prosthesis [34]. In patients diagnosed with aortic valve stenosis, mitral annular plane excursion is known to be reduced, while LVEF or other LV function parameters might be within normal values. However, it does seem to be more markedly reduced in symptomatic patients compared with asymptomatic; moreover, Takeda S et al. also noticed that it may be of use as a predictor of symptom onset in this subset of patients [35].

### 3.5. Intensive Care Unit

MAPSE is a valuable echocardiographic tool in ICU patients too, being reported in a number of articles to reflect both systolic and diastolic function in critically ill patients, while also correlating well with myocardial injury in patients with systemic inflammatory response syndrome (SIRS) [36]. MAPSE, along with TAPSE, LVEF. and lung ultrasound score (LUS score) seem to be significantly related to ICU mortality, according to a study published by Yin W et al. in 2017 [19]. Another significant advantage regarding its use in the ICU is that MAPSE assessment does not require good image quality or an experienced operator.

### 3.6. Arrhythmias

Reduced MAPSE, along with LAVI (left atrial volume index), seem to play a role in predicting atrial fibrillation (AF) recurrences after catheter ablation, according to one article, and may be used as a marker in the future, with more research being required on this topic [37].

### 3.7. Pediatric

MAPSE variations have been described as being due to cardiac size, which implies that its use in the pediatric population should be adjusted for body size. One study concludes that it can be reliably used in children and is most significantly associated with GLS [25,38]. It also seems that MAPSE is highly reproducible in inter-observer scenarios and that image quality has minimum influence on its measurement and thus can be a robust LV evaluation tool in children [6].

### 3.8. Oncology

A study performed on 40 patients by Lund M et al., published in 2015, set out to evaluate the effect on LV function of chemotherapy in the case of esophagus or gastroesophageal cancer. The results found that, in a neoadjuvant chemoradiotherapy group, there was a significant decrease in LV function determined by a significant decrease in septal MAPSE (mean change −1.4 mm, with a *p* value of 0.02) among other ultrasound parameters [23].

### 3.9. COVID-19

Given the recent COVID-19 pandemic and its emerging cardiovascular implications, several authors have set out to investigate possible correlations between SARS-CoV-2 infection and cardiac dysfunction. An article published in 2020 in the Journal of the American Society of Echocardiography by Jarori U et al. suggested that MAPSE in combination with right atrial (RA) size can be used to accurately stratify the risk in patients with severe COVID-19 infection, enabling them to be more appropriately triaged and more aggressively treated early on. MAPSE, according to the same study, seems to be the only left heart parameter that was independently associated with increased mortality in this context [12].

## 4. Discussion

There are limitations to MAPSE use and interpretation in certain specific cases, such as after cardiac surgery, significant pericardial effusions, mitral valve disease, or localized areas of motion abnormality or fibrosis, situations in which the use of this parameter should be more carefully applied, as its result might not accurately reflect longitudinal function. As far as cardiac pacing is concerned, the data seem scarce, with no such specific situation being investigated; therefore, this is an area in which more research might be carried out as far as validating MAPSE as a tool for LV appreciation in this context. An article published by Kai H et al. in late 2012 postulates that, in patients with paradoxical septal motion (PSM), also referred to as septal bounce, such as that seen in patients with left bundle branch block or conventional RV pacing, septal MAPSE might also reflect RV abnormalities (for which we have a similar M-mode parameter that has been historically used—tricuspid annular plane systolic excursion—TAPSE). They also recommend the use of lateral MAPSE for LV longitudinal function quantification [25,39].

In recent times, with the growth of cardiac magnetic resonance (CMR) imaging, MAPSE measurement in the four-chamber cine view has become a valuable index, comparable to echocardiography. Recently published articles have suggested that CMR-derived MAPSE is a predictor of mortality in hypertensive patients and independently predicts long-term prognosis following an ST-segment elevation myocardial infarction [40,41].

The major advantages of this simple tool are its good correlation with LVEF, its simple and excellent reproducibility between users, and the fact that it is mostly independent of image quality, while its disadvantages are connected to its limitations.

The limitations of MAPSE use are related to the fact that is an M-mode parameter and thus is angle-dependent and unable to detect regional wall abnormalities. There is also a caveat in its use in the case of pericardial effusions (especially large ones that cause mobile apex) and in mitral valve disease and calcifications of the mitral ring, in which MAPSE assessment should not be performed, as its direct measurement is not accurately possible.

### Gaps in Evidence and Future Perspectives

There is a lack of evidence on MAPSE usefulness in areas such as cardiac pacing and pacing-induced cardiomyopathy (PICM), where it might prove to be a useful tool for quick LV function assessment, thus justifying further research. Alatic J et al. published an article in 2022 on using MAPSE as a predictor of atrial fibrillation recurrence after pulmonary vein isolation (PVI), with the conclusion being presented in subchapter 3.6. In addition, due to the study limitations, more research is needed, as MAPSE could prove to be a diagnostic criterion in patients referred for catheter ablation of atrial fibrillation [37]. They also postulate that CMR-derived MAPSE might be used as a prognostic marker in patients with atrial fibrillation after PVI. Also, regarding CMR-derived MAPSE, there is increasing evidence of its utilization and usefulness in varying situations. A 2019 article by Romano et al. proved that its CMR-derived equivalent is a strong predictor of mortality in patients with hypertensive heart disease and that it might serve a role in identifying and stratifying the patients at a highest risk [40]. The same seems to be true in the case of acute coronary syndromes, where Mayr A et al. concluded that CMR MAPSE is a predictor of major adverse cardiovascular events (MACE) after an ST-elevation myocardial infarction, while also suggesting that more research is needed to establish a correlation between MAPSE and other markers such as left atrium parameters [41]. There is obviously room for ample research in the world of CMR-derived indices of LV longitudinal function, but we also believe that classic ultrasound M-mode-derived MAPSE still represents a field of research warranting more interest.

## 5. Conclusions

MAPSE seems to be a reliable and easy-to-use echocardiographic marker of LV function that not only correlates well with LVEF, but also proves to be extremely useful in the assessment of cardiac function in a wide array of clinical applications and might even have predictive value. Publications on its use in the last decade, however, seem to be insufficient, and it appears to be an underrepresented and underused LV function marker in comparison with others. There is a lack of evidence and validation regarding its use in certain specific cases such as, but not limited to, ventricular paced rhythms in patients with cardiac implantable electronic devices (CIED). It is a tried and tested ultrasound parameter that has proven to be applicable for the early detection of cardiac abnormalities, predicting the risk of heart failure and monitoring treatment response, especially in patients with poor acoustic windows.

## Figures and Tables

**Table 1:** Articles published in the last decade about MAPSE. Abbreviations: Pts no, patients number; MAPSE, mitral annular plane systolic excursion; LVEF, left ventricular ejection fraction; LVT, left ventricular thrombus; TAPSE, tricuspid annular plane systolic excursion; LUSS, lung ultrasound score; APACHE II, Acute Physiology and Chronic Health Evaluation II; RA, right atrium; LV, left ventricle; MS, multiple sclerosis; ICU, intensive care unit; HFpEF, heart failure with preserved ejection fraction; LVLS, left ventricle longitudinal strain; NTpro-BNP, N-terminal prohormone of brain natriuretic peptide.

Title	Authors	Year	Pts No	Study Type	Conclusion
Cardiac dysfunction in mixed connective tissue disease: a nationwide observational study	Berger S.G. et al. [9]	2023	136	Observational	MAPSE was affected in patients with mixed connective tissue disease.
Males with abdominal aortic aneurysm have reduced left ventricular systolic and diastolic function	Åström Malm I et al. [10]	2021	307	Observational	MAPSE was reduced in subjects with abdominal aortic aneurysm.
Echocardiographic risk factors of left ventricular thrombus in patients with acute anterior myocardial infarction	Chen M et al. [11]	2021	110	Case–control	Septal MAPSE was significantly lower in the LVT group than in the control group.
Mitral Annular Plane Systolic Excursion: An Early Marker of Mortality in Severe COVID-19	Jarori U et al. [12]	2020	68	Multi-center study	MAPSE emerged as the only left heart parameter independently associated with increased mortality.
Hemodynamics in Shock Patients Assessed by Critical Care Ultrasound and Its Relationship to Outcome: A Prospective Study	Zou T et al. [13]	2020	181	Prospective	MAPSE, S′-MV, TAPSE, LUSS, APACHE II, lactate, and PaO_2_/FiO_2_ were independently related to 28-day mortality, with MAPSE and S′-MV responding best for left heart function.
Left Ventricular Diastolic and Systolic Functions in Patients with Hypothyroidism	Tafarshiku R et al. [14]	2020	81	Observational	In patients with hypothyroidism and a reduced waste/hip ratio, there was significant evidence for compromised LV longitudinal systolic function in the form of long-axis amplitude of motion (MAPSE) and its systolic velocity.
Intraobserver and interobserver reproducibility of M-mode and B-mode acquired mitral annular plane systolic excursion (MAPSE) and its dependency on echocardiographic image quality in children	Hensel K O et al. [6]	2018	284	Observational	Echocardiographic image quality essentially has a negligible effect on MAPSE reproducibility and measurements. MAPSE is a robust echocardiographic parameter with convincing reproducibility for the assessment of LV function in children—even in patients with substandard imaging conditions.
Value of mitral annular plane systolic excursion in the assessment of contractile reserve in patients with ischemic cardiomyopathy before cardiac revascularization	G. Magdy et al. [15]	2018	50	Retrospective	MAPSE is a rapid, simple quantitative echocardiographic method that can assess contractile reserve in patients with ischemic cardiomyopathy before cardiac revascularization.
Evaluation of sepsis induced cardiac dysfunction as a predictor of mortality	Havaldar A.A. [16]	2018	58	Prospective	Sepsis-induced cardiac dysfunction assessed by echocardiography showed that the measurement of MAPSE when combined with APACHE II was a good predictor of mortality. Among the echocardiographic parameters, MAPSE alone was a good predictor of mortality.
Impaired Cardiac Function in Patients with Multiple Sclerosis by Comparison with Normal Subjects	Mincu R.I. et al. [17]	2018	103	Prospective	Patients with MS had decreased LV systolic function compared to the control subjects, as seen by lower 2D left ventricular ejection fraction (LVEF), lower 3D LVEF, MAPSE, lower “online” longitudinal myocardial systolic velocity (S’), lower global longitudinal strain (LS), and lower 3D LS.
Left Ventricular Longitudinal Systolic Function in Septic Shock Patients with Normal Ejection Fraction: A Case-control Study	Zhang H.M. et al. [18]	2017	90	Case–control	In the heart function appraisal of septic shock patients with a normal ejection fraction, more attention should be given to longitudinal function parameters such as MAPSE.
The utilization of critical care ultrasound to assess hemody-namics and lung pathology on ICU admission and the potential for predicting outcome	Yin W et al. [19]	2017	451	Retrospective	The TAPSE, ejection fraction (EF), MAPSE, and lung ultrasound score (LUS score) were significantly related to ICU mortality.
Left ventricular longitudinal systolic dysfunction is associated with right atrial dyssynchrony in heart failure with preserved ejection fraction.	Bytyçi I et al. [20]	2016	85	Observational	In patients with HFpEF and impaired MAPSE, RA dyssynchrony increased, compared to those with normal MAPSE.
Mitral annular plane systolic excursion in the assessment of left ventricular diastolic dysfunction in obese adults	Taşolar H et al. [21]	2015	80	Prospective	MAPSE may be useful in the stratification of left ventricle diastolic dysfunction in obese adults.
M-mode apical systolic excursion: A new and simple method to evaluate global left ventricular longitudinal strain	Amado J et al. [22]	2015	31	Observational	MAPSE and especially MMASE appear to be related to global LVLS.
Effects on heart function of neoadjuvant chemotherapy and chemoradiotherapy in patients with cancer in the esophagus or gastroesophageal junction—a prospective cohort pilot study within a randomized clinical trial	Lund M et al. [23]	2015	40	Prospective multi-center	Septal MAPSE decreased significantly in the chemoradiotherapy group.
Noninvasive Risk Stratification of Patients With Transthyretin Amyloidosis	Kristen A.V. et al. [24]	2014	70	Retrospective	NT-proBNP, troponin T, MAPSE, and the LV hypertrophy index were useful as predictors of outcome. MAPSE was predictive of survival.

## Data Availability

No new data were created or analyzed in this study. Data sharing is not applicable to this article.
